# Proton Transport in the Gadolinium-Doped Layered Perovskite BaLaInO_4_

**DOI:** 10.3390/ma15207351

**Published:** 2022-10-20

**Authors:** Nataliia Tarasova, Anzhelika Bedarkova, Irina Animitsa

**Affiliations:** 1 The Institute of High Temperature Electrochemistry of the Ural Branch of the Russian Academy of Sciences, 620660 Yekaterinburg, Russia; 2 Institute of Hydrogen Energy, Ural Federal University, 620000 Yekaterinburg, Russia

**Keywords:** BaLaInO_4_, layered perovskite, Ruddlesden-Popper structure, proton conductivity

## Abstract

Materials capable for use in energy generation have been actively investigated recently. Thermoelectrics, photovoltaics and electronic/ionic conductors are considered as a part of the modern energy system. Layered perovskites have many attractions, as materials with high conductivity. Gadolinium-doped layered perovskite BaLaInO_4_ was obtained and investigated for the first time. The high values of conductivity were proved. The composition BaLa_0.9_Gd_0.1_InO_4_ demonstrates predominantly protonic transport under wet air and low temperatures (<400 °C). The doping by rare earth metals of layered perovskite is a prospective method for significantly improving conductivity.

## 1. Introduction

Materials capable of being used in energy generation have been actively investigated recently [1,2,3,4,5]. Thermoelectrics [6,7,8,9], photovoltaics [10,11,12,13,14], and electronic/ionic conductors [15,16,17,18,19,20] are considered a part of the modern energy system. Layered perovskites have many attractions due to their different target properties, including solar energy utilization task [21,22,23,24] and production energy from chemical reactions. In recent decades, layered perovskites based on BaLaInO_4_ [25,26,27,28,29,30], SrLaInO_4_ [31,32,33,34,35], BaNdInO_4_ [36,37,38,39,40,41], BaNdScO_4_ [42] have been investigated as mixed ionic/electronic and protonic conductors. Features such as the crystal structure of these compositions define their prospective transport properties.

**Figure 1:**
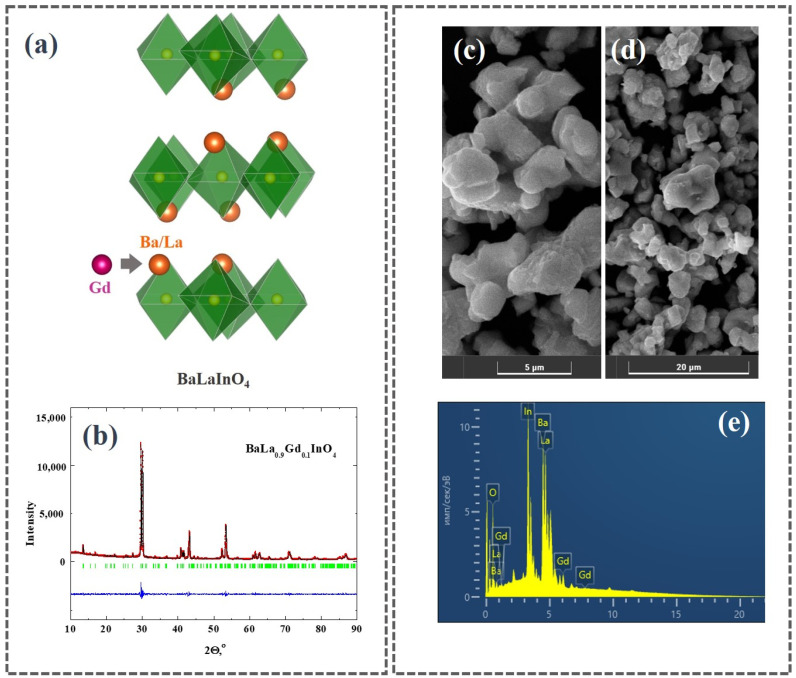
Scheme of gadolinium doping of BaLaInO_4_:(**a**), XRD-patterns (red dots are experimental data, black line is the theoretical fitting, blue line is the difference between the experimental and the calculated one after refinement, vertical green bars are Bragg angle positions) (**b**), SEM-images (**c**,**d**) and EDS-spectrum (**e**) of BaLa_0.9_Gd_0.1_InO_4_ composition.

The structure of classical perovskite ABO_3_ is a network of octahedra interconnected by vertices. Each oxygen atom is included into this network and interstitial oxygen atoms are absent in the structure. Layered perovskite AA′BO_4_ consists of perovskite layers [ABO_3_], in which octahedra are connected only by oxygen in the equatorial plane (Figure 1). Apical oxygen atoms are not connected to each other, which makes the structure more flexible to various substitutions. In addition, the oxygen atoms located in salt blocks [A′O] can be considered as interstitial oxygen atoms, relative to perovskite octahedra. These features can provide more mobility of oxygen atoms and an increase in the electrical conductivity during various types of substitutions.

The method of heterovalent substitution has proved to be successful as a way to design novel high-conductive materials with mixed ionic/electronic and protonic conductivity [43]. Isovalent substitution is a new prospective method for significant improvement of electrical conductivity. In this paper, the substitution of lanthanum sublattice by gadolinium ions was provided for the first time. Transport properties of BaLa_0.9_Gd_0.1_InO_4_ composition were investigated.

## 2. Materials and Methods

The composition BaLa_0.9_Gd_0.1_InO_4_ was synthesized using solid state method. The starting reagents BaCO_3_, In_2_O_3_, La_2_O_3_ and Gd_2_O_3_ (99.99% purity, REACHIM, Moscow, Russia) were used. The final temperature of calcination was 1300 °C.

The XRD investigations were performed using a Bruker Advance D8 Cu K*_α_* diffractometer (step of 0.01°, scanning rate of 0.5°/min). The morphology and chemical composition of the samples were studied using a VEGA3 TESCAN scanning electron microscope (SEM) equipped with a system for energy-dispersive X-ray spectroscopy (EDS). The investigation of the morphology and size of the grain of the powder samples were used. The determination of chemical composition was implemented on the ceramic samples.

The thermogravimetry (TG) was made using STA 409 PC Netzsch Analyser. The heating of initially hydrated samples was made at the temperature range of 40–1100 °C with the rate of 10 °C/min under a flow of dry Ar. The electrical conductivity was measured using impedance spectrometer Z-1000P, Elins, RF. The ceramic pellets were pressed for the investigations. The density was defined as the ratio of geometric and X-ray density and it was ~95%. The investigations were made from 1000 to 200 °C with 1°/min cooling rate under dry air or dry Ar conditions. The dry gas (air or Ar) was produced by circulating the gas through P_2_O_5_ (*p*H_2_O = 3.5·10^−5^ atm). The wet gas (air or Ar) was obtained by bubbling the gas at room temperature first through distilled water and then through saturated solution of KBr (*p*H_2_O = 2·10^−2^ atm).

## 3. Results

The results of the full-profile analysis of XRD-data are presented in Figure 1b. The obtained BaLa_0.9_Gd_0.1_InO_4_ composition belongs to the *Pbca* space group. The values of lattice parameters and unit cell volume are presented in Table 1. The introduction of Gd^3+^-cations into the La^3+^-sublattice with a slightly smaller ionic radii ($r_{{\mathrm{La}}^{3+}}$ = 1.216 Å, $r_{{\mathrm{Gd}}^{3+}}$ = 1.107 Å [44]) causes an increase in the size of the unit cell. The reason for the change in the interatomic distance during doping is the difference in the electronegativity of the elements. The introduction of ions with another electronegativity value into the cationic sublattice ($\chi_{\mathrm{La}}$ = 1.10, $\chi_{\mathrm{Gd}}$ = 1.20 [45]) can lead to the occurrence of additional repulsion effects. An increase in the interatomic distances and lattice parameters can be the result of this interaction. SEM-images of BaLa_0.9_Gd_0.1_InO_4_ composition with a different zoom are presented in Figure 1c,d. The sample consists of grains with an irregular shape and size around 5–10 µm. EDS analysis confirms the good agreement between theoretical and experimental element content (Table 2).

**Figure 2:**
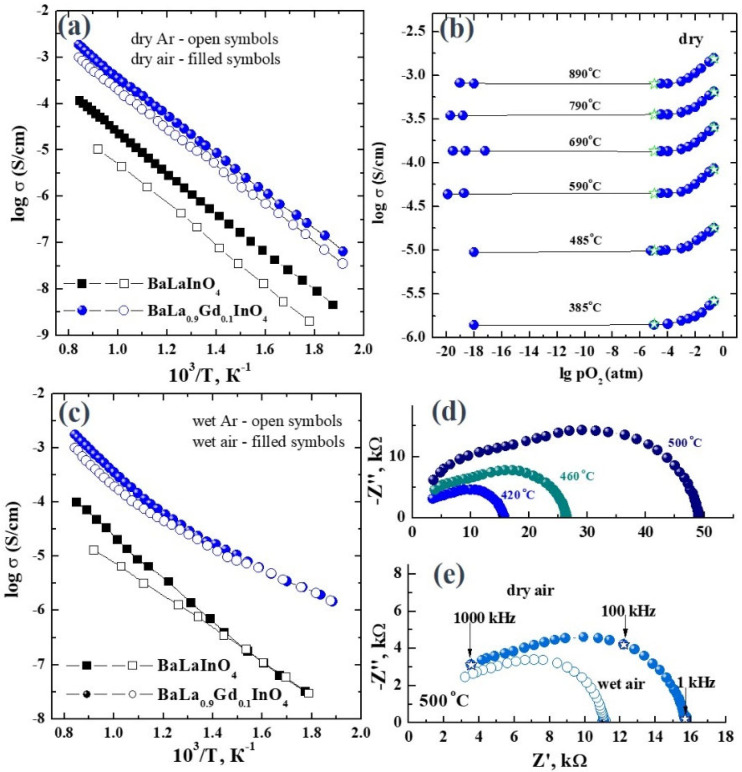
Temperature dependencies of conductivity for doped BaLa_0.9_Gd_0.1_InO_4_ and undoped BaLaInO_4_ compositions under dry (**a**) and wet (**c**) conditions; dependencies of conductivity vs. oxygen partial pressure (**b**); EIS-plots for BaLa_0.9_Gd_0.1_InO_4_ composition at different temperatures under dry air (**d**) and under dry and wet air (**e**) at 500 °C.

The investigation of the electrical properties was performed using impedance spectroscopy method. Figure 2d,e represents the examples of EIS-plots obtained at different temperatures (Figure 2d) and under different water partial pressure (Figure 2e). All plots contains two arcs. The first arc starts from zero coordinates and corresponds to the bulk resistance. The capacity of this arc is about ~10^−12^ F. The second arc takes place in the area of smaller frequencies and belongs to the resistance of grain boundaries. The capacity of the second arc has a magnitude of about ~10^−10^ F. The values of electrical conductivity were calculated using the bulk resistance values.

The dependencies of electrical conductivity vs. temperature obtained under dry conditions are presented in Figure 2a. The conductivity values of the doped composition are higher than the undoped by about 1.2 orders of magnitude in the whole temperature range. The isovalent doping does not change the concentration of oxygen point defects in the structure. Therefore, the only possible reason for conductivity increasing is the increase in the oxygen mobility. Gadolinium doping leads to the increase in the lattice parameters of unit cell, i.e., to the increase in the space for the ionic transfer.

The results on the effect of oxygen partial pressure on the conductivity are presented in Figure 2b. Under oxidizing conditions (pO_2_ > 10^−4^ atm), the conductivity curves have a positive slope, which indicates the mixed oxygen-ionic/hole conductivity nature:

(1) 12O2↔Oi″+2h•,

where $O_{i}^{\prime \prime }$ is the oxygen atom in the interstitial position, *h*^•^ is the hole.

Under reducing conditions (pO_2_ < 10^−4^ atm), the conductivity does not depend on the oxygen partial pressure and corresponds to the oxygen-ionic conductivity. The conductivity values obtained under dry Ar (green symbols in Figure 2b) are very close to the conductivity values obtained at pO_2_ = 10^−5^ atm and they can be considered as oxygen-ionic conductivity values. The calculation of oxygen-ionic transport numbers was made as the relation of conductivity values obtained under dry Ar and dry air conditions:

(2) $$t_{O^{2-}}=\frac{\sigma_{\mathrm{dry}\;\mathrm{Ar}}}{\sigma_{\mathrm{dry}\;\mathrm{air}}}$$

The oxygen-ionic transport numbers for both doped BaLa_0.9_Gd_0.1_InO_4_ and undoped BaLaInO_4_ compositions are constant throughout all the investigated temperature range. At the same time, the values are higher for doped compositions and they reach 45% compared to 20% for the undoped sample. Thus, gadolinium doping provides the increase in the electrical conductivity values and the oxygen-ionic transport numbers at the same time.

**Figure 3:**
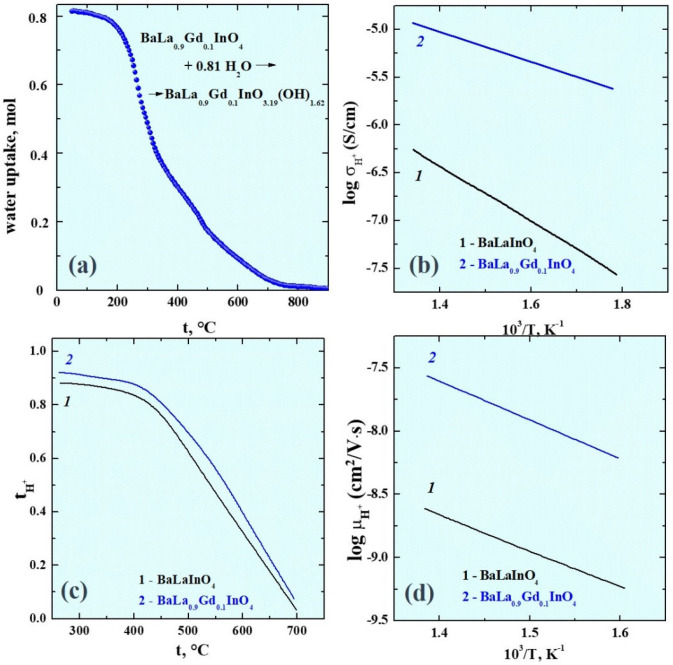
TG-curve for BaLa_0.9_Gd_0.1_InO_4_ composition (**a**); temperature dependencies of protonic conductivity (**b**) and mobility (**d**) and protonic transport numbers (**c**) for doped BaLa_0.9_Gd_0.1_InO_4_ and undoped BaLaInO_4_ compositions.

The effect of the water partial pressure on the electrical conductivity was also investigated. The conductivity vs. temperature dependencies for doped BaLa_0.9_Gd_0.1_InO_4_ and undoped BaLaInO_4_ samples are represented in Figure 2c. The conductivity values of the doped composition are higher, by up to two orders of magnitude at 300 °C, compared to the undoped sample. The effect of atmosphere humidity starts from ~700 °C, which is proved by thermogravimetric measurements (Figure 3a). The decrease in the temperature leads to the increase in the proton concentration in the structure due to the dissociative intercalation of water molecules into the interlayer space of layered perovskite:

(3) $$H_{2}O+O_{O}^{x}↔{(\mathrm{OH})}_{O}^{•}+{\left(\mathrm{OH}\right)}_{i}^{\prime }$$

where ${(\mathrm{OH})}_{o}^{•}$ is the hydroxyl group in the regular oxygen position; ${\left(\mathrm{OH}\right)}_{i}^{\prime }$ is the hydroxyl group located in the interlayer space. Water uptake reaches 0.81 mol water per formula unit for doped composition. It is greater than for the undoped composition (0.62 mol [43]), which can be explained by the increase in the unit cell volume of the doped composition. The interaction of protons with holes can be expressed as:

(4) 2h•+12H2O + Oo×↔14O2+(OH)O•

where *h*^•^ is the hole, ${(\mathrm{OH})}_{o}^{•}$ is the hydroxyl group in the regular oxygen position, $O_{o}^{\times }$ is the oxygen atom in a regular position.

This interaction leads to the decrease in the holes concentration and increase in the share of ionic transport. The conductivity values obtained under wet air and wet Ar are very close to each other, below 400 °C, which indicates the dominance of ionic conductivity at low temperatures.

Protonic conductivity values were calculated as the difference between conductivity values in wet Ar and dry Ar. The curves of protonic conductivity vs. temperature for doped and undoped compositions are presented in Figure 3b. The protonic conductivity values of BaLa_0.9_Gd_0.1_InO_4_ composition are higher than BaLaInO_4_ composition by about two orders of magnitude at 300 °C. This can be due to both an increase in the concentration of protons and their mobility. The proton mobility was calculated according to the equation:

(5) $$\mu_{H^{+}}=\frac{\sigma_{H^{+}}}{e\cdot c_{H^{+}}}$$

The temperature dependencies of proton mobility for doped and undoped compositions are presented in Figure 3d. The doping provides the increase in proton mobility, by about one order of magnitude. The most probable reason is the increase in the size of the unit cell and the space for the ionic transport. Thus, the gadolinium doping leads to the increase in the concentration and mobility of protons in the structure of layered perovskite.

The protonic transport numbers were calculated using the formula:

(6) $$t_{p}=\frac{\sigma_{wet\;Ar}-\sigma_{dry\;Ar}}{\sigma_{wet}}$$

The curves of protonic transport numbers vs. temperature for doped and undoped compositions are presented in Figure 3c. They are about 95% at the temperatures below 400 °C for both of these compositions. In other words, they demonstrate predominantly protonic transport under wet air and low (T < 400 °C) temperatures. The substitution of lanthanum sublattice by gadolinium ions provides a significant increase in the conductivity values. Obtained BaLa_0.9_Gd_0.1_InO_4_ composition demonstrates 6.3·10^−6^ S/cm protonic conductivity value at 400 °C, compared with 2.7·10^−7^ S/cm for undoped BaLaInO_4_ composition.

## 4. Conclusions

In this paper, the substitution of lanthanum sublattice by gadolinium ions was provided for the first time. The layered composition BaLa_0.9_Gd_0.1_InO_4_ was obtained using solid state method. The possibility for hydration was proved by thermogravimetry method. Electrical conductivity was measured under variation in temperature and water and oxygen partial pressures. It was proved that protonic conductivity values of BaLa_0.9_Gd_0.1_InO_4_ composition are higher than BaLaInO_4_ composition, by about two orders of magnitude at 300 °C. Composition BaLa_0.9_Gd_0.1_InO_4_ demonstrates predominantly protonic transport under wet air and low temperatures. The doping by rare earth metals of layered perovskite is a prospective method for significantly improving conductivity.

## Figures and Tables

**Table 1:** The parameters of unit cell for compositions BaLaInO_4_ and BaLa_0.9_Gd_0.1_InO_4_.

Composition	*a*, Å	*b*, Å	*c*, Å	Unit Cell Volume, (Å^3^)
BaLaInO_4_	12.932(3)	5.906(0)	5.894(2)	450.19(5)
BaLa_0.9_Gd_0.1_InO_4_	12.988(5)	5.908(1)	5.910(8)	453.92(8)

**Table 2:** The average element ratios determined by EDS analysis for the sample BaLa_0.9_Gd_0.1_InO_4_ (theoretical values are in brackets).

Element	Ba	La	Nd	In
Content, atomic %	33.5 ± 0.7 (33.3)	29.8 ± 0.6 (30.0)	3.2 ± 0.1 (3.3)	33.5 ± 0.7 (33.4)

## Data Availability

Not applicable.
